# Gunshot-Induced Popliteal Artery Pseudoaneurysm: A Case Report

**DOI:** 10.7759/cureus.85660

**Published:** 2025-06-09

**Authors:** Elizabeth Alcala, José Manuel García Romero, Pedro Hugo Guerrero Morales, Daniela De Noriega Guzmán, Alejandro Morales Rubio

**Affiliations:** 1 Surgery, Hospital General de Querétaro, Querétaro, MEX; 2 Surgery, Hospital Zambrano Hellion TecSalud, San Pedro Garza García, MEX

**Keywords:** gunshot wound, popliteal artery, pseudoaneurysm, traumatic vascular injury, vascular trauma

## Abstract

Popliteal artery injuries are uncommon but carry a high risk of limb-threatening complications. Among these, pseudoaneurysms caused by penetrating trauma, such as gunshot wounds, are particularly rare and clinically significant due to their potential for delayed presentation and high morbidity. We report the case of a 46-year-old male who sustained a gunshot wound to the left thigh. CT angiography revealed a pseudoaneurysm with >50% transection of the popliteal artery. Urgent surgical repair with thrombectomy and polytetrafluoroethylene graft placement led to full neurovascular recovery. This case underscores the limitations of relying solely on clinical examination to rule out vascular injury, as distal pulses may remain intact despite significant arterial compromise. Clinicians should maintain a high index of suspicion and promptly employ imaging, especially CT angiography, in any equivocal or evolving limb trauma. The delayed onset of ischemic and neurological signs highlights the importance of maintaining a high index of suspicion and utilizing CT angiography in equivocal cases. Prompt recognition and surgical intervention, supported by advances in imaging techniques and vascular graft materials, are key to preventing irreversible damage and optimizing limb salvage outcomes.

## Introduction

The popliteal artery, a continuation of the superficial femoral artery, courses through the popliteal fossa behind the knee joint and provides critical blood supply to the lower leg via its terminal branches (the anterior and posterior tibial arteries) [1]. Its deep anatomical position near bony structures and joints makes it particularly susceptible to both traumatic and iatrogenic injuries, making careful assessment crucial in clinical practice. Failure to promptly identify and manage such injuries can lead to limb-threatening ischemia, compartment syndrome, or even amputation [2].

Although traumatic injuries to the popliteal artery are relatively rare, constituting about 0.2% of all traumas and 5% of vascular injuries, they are significantly associated with knee dislocations, complicating approximately 16% of such cases [2]. If not promptly diagnosed and managed, these injuries can result in serious complications such as limb ischemia, amputation, and permanent disability. Historically, amputation rates for missed or late-diagnosed cases were as high as 70%. However, recent advancements have reduced this to around 20%, underscoring the critical need for early detection and timely intervention [2].

Vascular injuries of the lower extremity, particularly those involving the popliteal artery, present a complex clinical challenge due to their association with high rates of limb loss and significant morbidity. Accurate assessment and timely management are critical for improving outcomes. Several scoring systems have been developed to assess limb viability and guide treatment decisions in vascular extremity trauma. The Mangled Extremity Severity Score (MESS) is a widely used historical tool that incorporates factors such as skeletal and soft tissue injury, limb ischemia, shock, and age, with scores of 7 or higher suggesting the need for amputation [3]. However, its predictive accuracy in modern clinical settings has been questioned. The PROOVIT registry (Prospective Vascular Injury Treatment) serves as a multicenter database for vascular trauma outcomes; however, it does not provide a standardized scoring system [3].

More recently, the POPSAVEIT score (Popliteal Scoring Assessment for Vascular Extremity Injuries in Trauma) was developed to provide a focused and validated method to assess vascular extremity injuries specifically at the popliteal region [3]. These tools are simple and clinically applicable, incorporating preoperative variables such as ischemia severity, fracture patterns, soft tissue damage, and associated nerve injuries into a cumulative score that aids early decision-making and helps predict the risk of limb loss [3]. Notably, the presence of peripheral pulses does not rule out a vascular injury [4].

Traumatic popliteal vascular injuries present a clinical challenge, as they are associated with the highest risk of limb loss among all peripheral vascular injuries, with major amputation rates ranging from 14% to 25% in the general population [5]. Popliteal artery injury accounts for approximately 14% of vascular injuries in the lower limb [3]. A pseudoaneurysm, or false aneurysm, occurs when there is a breach in the arterial wall, leading to the formation of a contained hematoma that communicates with the arterial lumen. This differs from a true aneurysm, which involves all three layers of the vessel wall [6]. Popliteal artery pseudoaneurysms are rare but potentially limb-threatening complications. They may arise due to various causes, including penetrating trauma (such as gunshot wounds), orthopedic or vascular surgical procedures, knee dislocations, or even repeated minor trauma [6].

Although the true incidence of popliteal artery pseudoaneurysms remains unclear due to their rarity, timely recognition is crucial for effective management and treatment. Delayed diagnosis may result in limb ischemia, compartment syndrome, thrombosis, or rupture, underscoring the importance of high clinical suspicion in patients presenting with progressive pain, swelling, or distal vascular compromise following trauma [3,6]. CT angiography is recommended as the first-line diagnostic tool in hemodynamically stable patients [7].

Herein, we report the case of a 46-year-old man who developed a popliteal artery pseudoaneurysm two days after sustaining a gunshot wound to the left thigh. This case illustrates how pseudoaneurysms can present with delayed and subtle symptoms, which may obscure the diagnosis and delay definitive care. Given the risk of rapid expansion, rupture, or distal ischemia, even short delays, as in this case, within 48 hours, can pose significant threats to limb viability. This underscores the critical importance of maintaining a high index of suspicion in penetrating extremity trauma and initiating timely imaging and surgical intervention.

## Case presentation

A 46-year-old Hispanic male patient, with a relevant past medical history of tobacco use (20 pack-years) and weekly crystal methamphetamine use over the past two years, presented to the emergency department after sustaining a gunshot wound with an entry point on the proximal anterolateral aspect of the left thigh and an exit wound on the distal posteromedial aspect of the same limb. The injury occurred approximately two hours prior during an interpersonal altercation and involved a low-caliber firearm (exact type unknown). On initial assessment, his vital signs included a heart rate of 120 bpm, blood pressure of 100/70 mmHg, oxygen saturation of 92% on room air, and cold peripheries. He was found to have palpable but weak distal pulses, progressive, intense burning pain, and increased diameter of the affected limb, raising concern for compartment syndrome and vascular compromise. Intracompartmental pressure was measured at 45 mmHg (delta pressure of 25 mmHg), further supporting the suspicion of developing compartment syndrome.

Following evaluation by the vascular surgery team, a fasciotomy and exploration of the femoral vessels were performed. No direct vascular injury was identified intraoperatively, and distal pulses improved over the following hours.

Twenty-four hours later, while in the surgical ward, the patient developed an absence of distal pulses, along with signs of localized ischemia, including generalized pallor, coldness, numbness of the limb, and throbbing pain. A second fasciotomy was performed on the anterolateral aspect of the left leg, resulting in improvement of the popliteal pulse; however, the dorsalis pedis pulse remained diminished, and skin pallor persisted.

**Figure 1:**
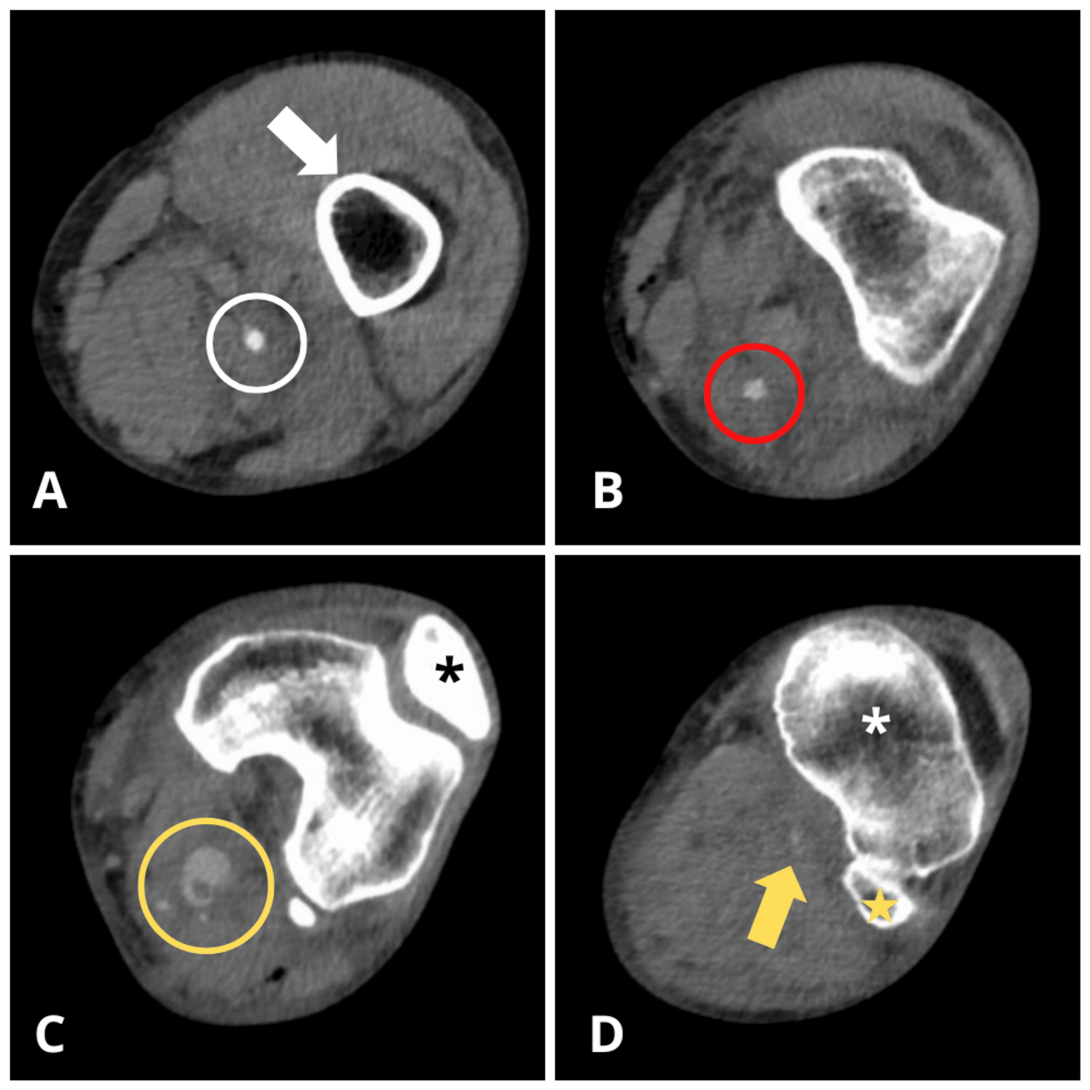
Contrast-enhanced axial CT images of the left leg showing the anatomical course and pathology of the popliteal artery (A) Origin of the left popliteal artery (white circle) as a continuation of the superficial femoral artery after passing through the adductor hiatus, coursing along the posteromedial surface of the distal femoral epiphysis (white arrow). (B) Left popliteal artery in its P1 segment (red circle), demonstrating a course posterior to the supracondylar portion of the femur, extending from the passage through the adductor hiatus to the superior border of the patella. (C) Left popliteal artery in its P2 segment, following an intercondylar retropatellar course as it passes posterior to the knee joint. A saccular image consistent with a pseudoaneurysm (yellow circle) is also visualized at the same axial level as the patella (black asterisk). (D) Left popliteal artery in its P3 segment at the level of the proximal tibial (white asterisk) and fibular (yellow star) articular condyles, demonstrating a marked reduction in blood flow, as evidenced by relative hypodensity of the vessel (yellow arrow) compared to the more proximal segments. CT: computed tomography

Due to persistently diminished distal pulses, ongoing pain despite adequate analgesia, and neurological symptoms including paresthesia, numbness, and weakness, a contrast-enhanced CT scan was performed (Figure 1).

**Figure 2:**
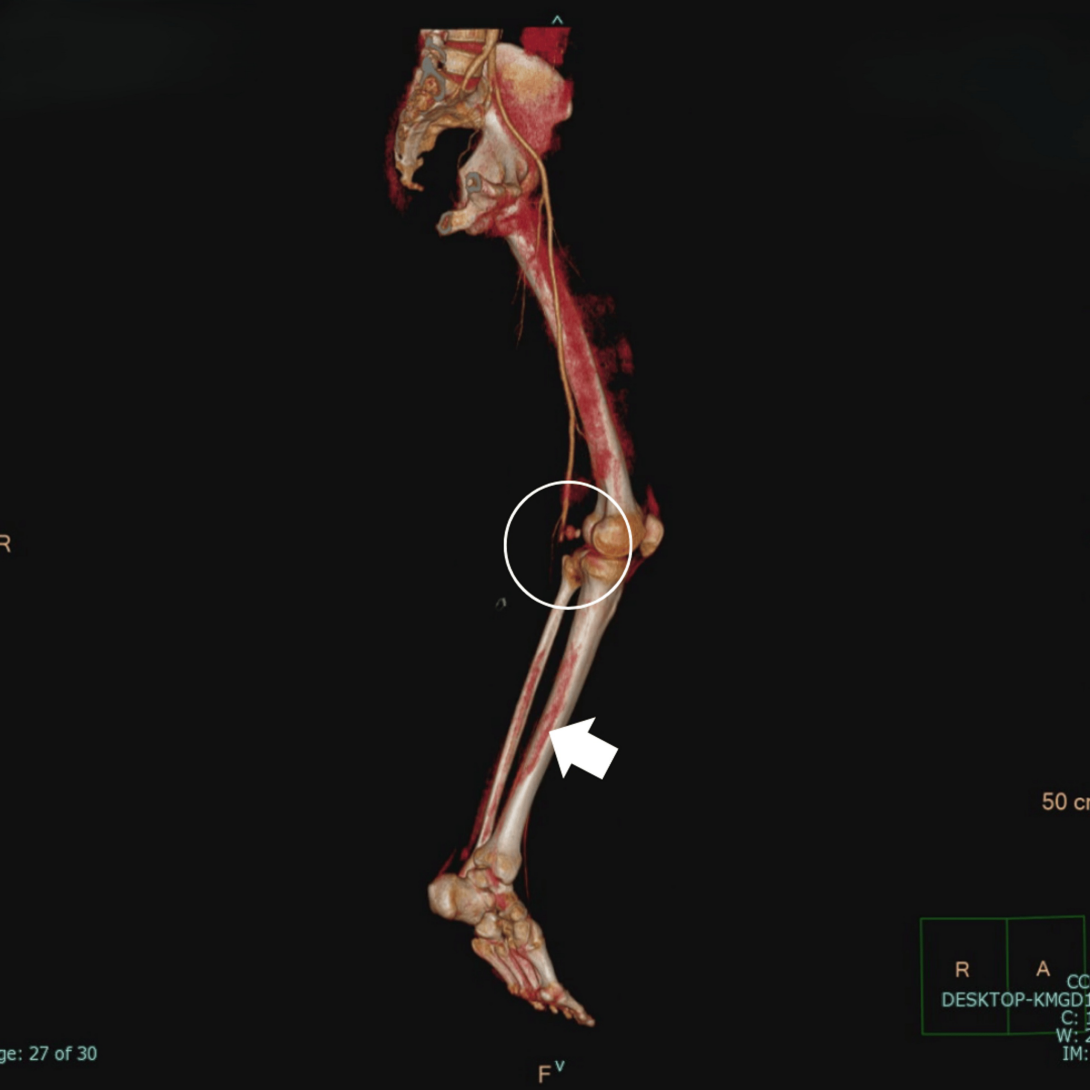
CT angiography showing distal popliteal artery injury with collateral perfusion CT angiography with vascular reconstruction demonstrated vascular compromise with pseudoaneurysm formation at the P2 segment (retroarticular), extending into the P3 segment (proximal tibial), resulting in an absence of distal flow in the popliteal artery, findings suggestive of vascular injury at this level (white circle). The tibioperoneal trunk was not visualized; however, faint contrast enhancement was observed in the distal vascular beds, indicating limited perfusion likely sustained via collateral arterial pathways (white arrow). CT: computed tomography

CT angiography confirmed the loss of arterial flow at the popliteal level, with marked distortion suggestive of a pseudoaneurysm (Figure 2).

**Figure 3:**
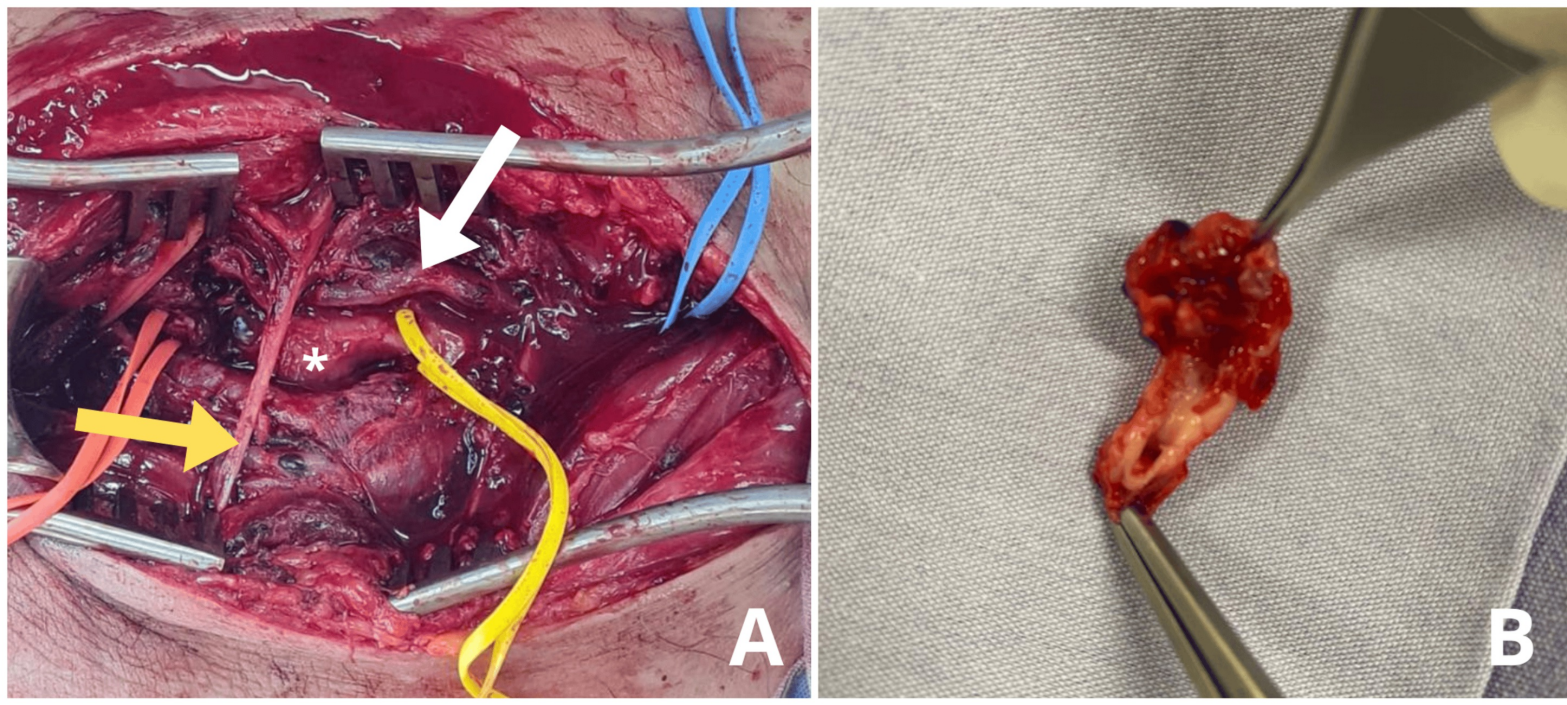
Intraoperative identification of popliteal pseudoaneurysm, tibial nerve branch, and popliteal vein (A) A posterior surgical approach was employed, providing optimal exposure and effective vascular control. Intraoperatively, a pseudoaneurysm of the popliteal artery involving the P2 and P3 segments was identified. Distal control was achieved just proximal to the tibioperoneal trunk using an orange vessel loop, while proximal control was obtained with a blue vessel loop. The pseudoaneurysm is visible (white asterisk). The popliteal vein (white arrow), crossing the neurovascular bundle, was intact. A branch of the tibial nerve (yellow arrow) was also clearly identified. (B) The resected segment of the popliteal artery demonstrates a 50% transection of the posterior vessel wall with hematoma infiltration of the adventitia.

The patient was taken to the vascular surgery department, where surgical exploration confirmed a popliteal artery pseudoaneurysm with >50% arterial transection and hematoma infiltration into surrounding musculature (Figure 3).

To evaluate the possibility of using an autologous venous graft, Doppler ultrasound mapping of the superficial venous system of the contralateral leg and both arms was performed. However, no suitable veins with a diameter greater than 2.5 mm were identified.

A surgical thrombectomy was performed, followed by the placement of a 7-4 mm × 70 cm IMPRA expanded polytetrafluoroethylene (PTFE) vascular graft. Adequate distal perfusion was confirmed using Doppler monitoring. The surgical thrombectomy involved the use of a 3 Fr, 40 cm Fogarty catheter for the tibioperoneal trunk and subsequently a 5 Fr, 80 cm Fogarty catheter for the popliteal artery, resulting in the extraction of multiple thrombi. The procedure was successful, with satisfactory backflow observed following the thrombectomy.

During the following week, a vacuum-assisted closure system was applied to the thigh fasciotomy site, achieving near-complete wound approximation. A final neurovascular assessment of the left lower limb showed no abnormalities or evidence of long-term compromise.

## Discussion

Popliteal artery injuries from penetrating trauma pose a significant diagnostic challenge, often leading to missed vascular damage and delayed complications such as pseudoaneurysm formation and neurological compromise. In our case, the patient initially retained distal pulses and showed no evidence of overt vascular injury during surgical exploration. However, delayed ischemic signs and neurological symptoms eventually revealed a pseudoaneurysm with >50% arterial transection [8].

In the series by Roganović et al., 22 patients with missile-induced pseudoaneurysms were studied, of whom nearly half presented with delayed neurological deterioration due to compressive effects of the expanding hematoma sac. Similarly, in our case, the patient initially had palpable distal pulses and no overt vascular injury, which delayed the diagnosis. This correlates with the finding that distal pulses may remain palpable in up to 25% of patients despite the presence of a pseudoaneurysm [6].

This pattern of delayed recognition is further highlighted in a separate study involving a military cohort. Yilmaz et al. conducted a retrospective review of 40 military patients with missed arterial injuries, noting a median diagnostic delay of 60 days. Interestingly, in their cohort, distal pulses were present in all but four patients despite the presence of serious vascular lesions, including false aneurysms and arteriovenous fistulas in over 85% of cases [8]. Our patient, though civilian, mirrored this pattern with preserved pulses early on, highlighting the danger of relying solely on clinical examination.

Notably, the patient in our case developed progressive neurological deficits (numbness, paresthesia) consistent with nerve compression from the expanding pseudoaneurysm, paralleling the neurological presentations in Roganović's cohort [6]. In that study, the duration of symptomatic compression was found to be a critical prognostic factor: patients with >3.5 days of nerve compression had significantly worse outcomes and often required nerve grafts. Prompt recognition and surgical intervention, including thrombectomy and synthetic graft placement, led to full neurovascular recovery in our patient, suggesting that timely action likely prevented irreversible nerve damage.

Another important consideration is the choice of graft material. Although autologous vein grafts are the first choice, there are situations where their use is not feasible, such as poor-quality saphenous veins, time-critical ischemia, or contaminated surgical fields [2,6]. In such cases, the decision to use a prosthetic graft is guided by the patient's condition, the urgency of revascularization, and the intraoperative assessment of available conduits. Synthetic grafts such as PTFE, ringed PTFE, and Dacron have been successfully used as alternatives [4]. Roganović et al. reported favorable outcomes with PTFE grafts, particularly in anatomically challenging areas or previously infected sites [6,8].

Given that autologous veins smaller than 2-3 mm in diameter are associated with reduced long-term graft patency, a ringed synthetic graft was selected as the most appropriate option for arterial reconstruction [4,9]. According to Dorigo et al., autologous venous grafts offer superior long-term patency compared to PTFE grafts; however, this advantage is contingent upon the availability of suitable venous tissue, such as a saphenous vein with a diameter greater than 3 mm, which was not present in our patient, possibly due to his long-standing smoking history [9]. In this case, a PTFE graft was successfully used to repair the popliteal artery, with no evidence of infection or graft-related complications during follow-up. These outcomes support the conclusion that synthetic grafts, such as PTFE, remain a viable and durable alternative in urgent vascular reconstruction when adequate autologous veins are unavailable.

The findings in our case align with those reported by Woolgar et al., who reviewed seven cases of delayed presentation of traumatic popliteal artery pseudoaneurysms. In their series, all patients suffered penetrating trauma (six gunshot wounds, one stab wound) and presented with large pseudoaneurysms after a median delay of 1.5 months [10]. Notably, despite the presence of sizeable pseudoaneurysms (all >8 cm), distal pulses remained palpable in every patient, mirroring our patient's preserved distal perfusion and underscoring the pitfall of relying solely on pulse presence to exclude vascular injury. Additionally, neurological deficits were common in their cohort, with four of seven patients presenting with varying levels of nerve dysfunction, including foot drop and total motor/sensory loss, findings that echo the progressive neurological deterioration seen in our patient [10,11]. While Woolgar's patients had more prolonged delays and often developed fixed flexion deformities of the knee, our patient avoided such orthopedic sequelae due to earlier intervention. This contrast emphasizes the value of timely imaging and surgical management in preventing permanent functional impairment.

The critical importance of prompt revascularization is further supported by a large retrospective study by Alarhayem et al., which analyzed 4,406 cases of lower extremity arterial injuries from the National Trauma Data Bank. The authors found that patients undergoing revascularization within 60 minutes of injury had significantly lower amputation rates (6%) compared to those treated after one to three hours (11.7%) and three to six hours (13.4%). Although our patient underwent surgery beyond the one-hour window, timely surgical intervention and thrombectomy with graft placement were likely key factors in preventing limb loss. Alarhayem et al. found that nerve injury and popliteal artery involvement significantly increased amputation risk [7]. In our patient, neurological symptoms developed alongside a popliteal artery pseudoaneurysm, reinforcing the severity of the presentation. The study's focus on rapid transport and early operative repair aligns with our case outcome, emphasizing that a high index of suspicion and expedited vascular imaging (CT angiography) are essential to minimize irreversible damage [7].

In addition to clinical judgment, scoring systems provide structured frameworks for evaluating limb-threatening vascular injuries (Table 1). While these tools offer valuable guidance, their utility may be limited by factors such as variability in clinical interpretation, limited validation in diverse civilian trauma populations, and the potential oversimplification of complex clinical scenarios [3]. They should, therefore, be considered as adjuncts to, rather than replacements for, a thorough clinical assessment.

**Table 1:** Comparison of vascular trauma scoring systems This table summarizes key vascular trauma scoring systems and registries, outlining their purpose, criteria, predictive thresholds for amputation (where applicable), strengths, and limitations. It highlights POPSAVEIT for popliteal artery injuries, MESS for general limb trauma, and PROOVIT as a data registry rather than a predictive tool [3]. POPSAVEIT: Popliteal Scoring Assessment for Vascular Extremity Injuries in Trauma, MESS: Mangled Extremity Severity Score, PROOVIT: Prospective Vascular Injury Treatment Registry, N/A: not a predictive score

Scoring system	Purpose	Scoring criteria	Amputation prediction threshold	Strengths	Limitations
POPSAVEIT	Predict the preoperative risk of amputation in popliteal artery injuries	Systolic blood pressure <90 mmHg (1 point); associated orthopedic injury (2 points); absence of pedal Doppler signals (2 points), or absence of pulse if Doppler is unavailable (1 point)	≥3 points = high risk of amputation (sensitivity 85%, specificity 49%)	Simple, focused on popliteal injuries	Limited to popliteal trauma; Doppler access not universal
MESS	Predict the need for primary amputation in severe limb trauma	Skeletal/soft tissue injury (1–4 points), limb ischemia (1–3 points; score is doubled if ischemia exceeds 6 hours), shock (0–2 points), and age (0–2 points)	≥7 points predict likely amputation	Historical relevance, comprehensive	Less predictive in modern settings, not validated for functional outcome
PROOVIT	Registry for vascular injury outcomes; not a scoring tool per se	Prospective data on vascular injuries from multiple trauma centers	N/A	Real-world, multicenter data source informs treatment trends	Not a risk stratification score; observational nature

Although the POPSAVEIT score is designed to aid in the preoperative risk stratification of traumatic popliteal artery injuries, its application in our case illustrates some of its limitations. Our patient received a score of 1, based solely on the absence of palpable pedal pulses without preoperative Doppler assessment, placing him in the low-risk category for amputation [3]. However, this score failed to capture the clinical severity of the case, which included compartment syndrome, progressive ischemia, and a popliteal artery pseudoaneurysm with significant arterial transection. As defined by the scoring system, orthopedic injuries are limited to fractures or dislocations, thereby excluding relevant musculoskeletal complications such as compartment syndrome. This case reinforces that while POPSAVEIT can be a useful adjunct for early triage, it should not be used in isolation, particularly in evolving or complex clinical scenarios where timely surgical judgment remains paramount.

Demographically, our patient shares several characteristics with the typical POPSAVEIT study cohort, including male sex and isolated popliteal artery involvement, specifically affecting segments P2 and P3, common locations in the original study. He is, however, older than the mean age reported (46 vs. 33 years), though age was not identified as a predictor of amputation. His initial systolic blood pressure was >90 mmHg (100/70 mmHg), which is associated with a lower risk of amputation according to the POPSAVEIT study. The absence of a palpable pedal pulse, without Doppler evaluation, contributed 1 point on the POPSAVEIT score, placing the patient in the low-risk category (0-2 points), where the amputation rate was only 5.9%. Additionally, the patient had a MESS of 3, receiving 1 point for a low-energy mechanism (gunshot wound), 1 point for reduced pulses but preserved perfusion, 0 points for being normotensive, and 1 point for age (30-50 years). This score is lower than the median MESS of 5 reported in the study, further supporting a lower injury severity. The patient's outcome with limb salvage is consistent with these predictive scores. However, his clinical course, with evolving ischemia and compartment syndrome, illustrates the necessity of ongoing clinical assessment beyond initial scoring tools.

The association between arterial transection and risk of limb loss was also highlighted in a large civilian series by Hafez et al., involving 550 patients with lower limb arterial injuries. In their multivariate analysis, arterial transection (OR: 2.8), compartment syndrome (OR: 4.1), compound fractures (OR: 2.7), and occluded grafts (OR: 16.7) emerged as significant independent predictors of amputation [5]. Our case reflects several of these risk factors: a greater than 50% arterial transection of the popliteal artery, clinical signs of compartment syndrome, and the need for graft placement. Fortunately, graft patency was maintained, likely contributing to the successful limb salvage. Their findings underscore the importance of prompt recognition, aggressive compartment management, and reliable revascularization in minimizing limb loss.

Finally, this case highlights the importance of maintaining a high index of suspicion for vascular injury, even when initial signs are equivocal. Delayed diagnosis often stems from initially preserved distal pulses, which can lead to false reassurance and missed early intervention. Our case illustrates how evolving vascular compromise may not be clinically apparent on initial examination, underscoring the critical role of CT angiography as a timely and definitive diagnostic tool in suspected arterial injuries.

## Conclusions

Failure to recognize vascular injury early can result in catastrophic consequences, including limb loss. This case underscores the importance of maintaining a high index of suspicion for such injuries in patients with penetrating trauma, even when distal pulses are initially present. The presence of palpable pulses does not reliably exclude significant arterial damage such as pseudoaneurysm or transection.

CT angiography played a decisive role in revealing the delayed vascular injury, offering crucial guidance for timely surgical intervention. Its routine use in cases of suspected arterial trauma can minimize missed diagnoses and significantly improve patient outcomes. When autologous vein grafts are not feasible, synthetic grafts, such as PTFE, remain a viable and effective alternative for revascularization, especially in urgent or anatomically complex scenarios. Ultimately, early recognition and decisive surgical management of vascular injuries are fundamental in preventing irreversible neurovascular compromise and maximizing limb salvage, even when initial findings appear reassuring.
